# Supplementary material for: Single-cell transcriptomic analysis reveals the association of Ccl6+Ccr2+Arg1+ macrophages with renal interstitial fibrosis in AKI
Source: PLoS One. 2025 Sep 15;20(9):e0332026. doi: 10.1371/journal.pone.0332026 (PMC12435735; doi:10.1371/journal.pone.0332026)
Supplement: S1 Fig — (PDF) [file pone.0332026.s001.pdf]

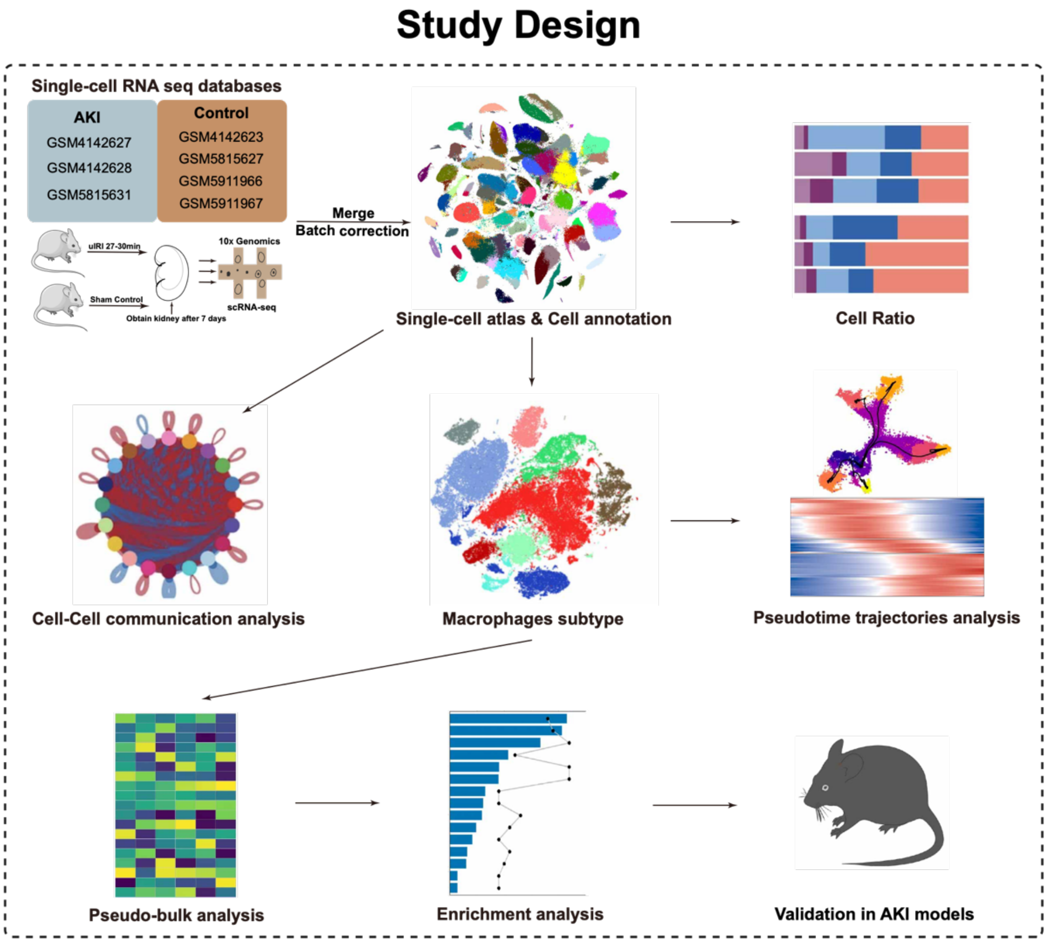

Supplementary Fig. 1 Flowchart of this study.

After integrating multiple single-cell transcriptomic datasets of AKI, batch effect removal, dimensionality reduction, and clustering were performed, followed by annotation to generate a single-cell atlas of the kidney at 7 days post-AKI onset. Cell composition analysis and intercellular interaction analysis were then conducted. Macrophage subpopulations were further selected for dimensionality reduction and clustering. Pseudotime analysis was subsequently performed on these macrophage subpopulations, and gene co-expression patterns during macrophage differentiation were examined. Additionally, enrichment analysis was conducted on genes upregulated in macrophage subpopulations in the context of AKI. Finally, these findings were validated using the uIRI-induced AKI model.
