# Supplementary material for: Single-cell transcriptomic analysis reveals the association of Ccl6+Ccr2+Arg1+ macrophages with renal interstitial fibrosis in AKI
Source: PLoS One. 2025 Sep 15;20(9):e0332026. doi: 10.1371/journal.pone.0332026 (PMC12435735; doi:10.1371/journal.pone.0332026)
Supplement: S2 Fig — (PDF) [file pone.0332026.s002.pdf]

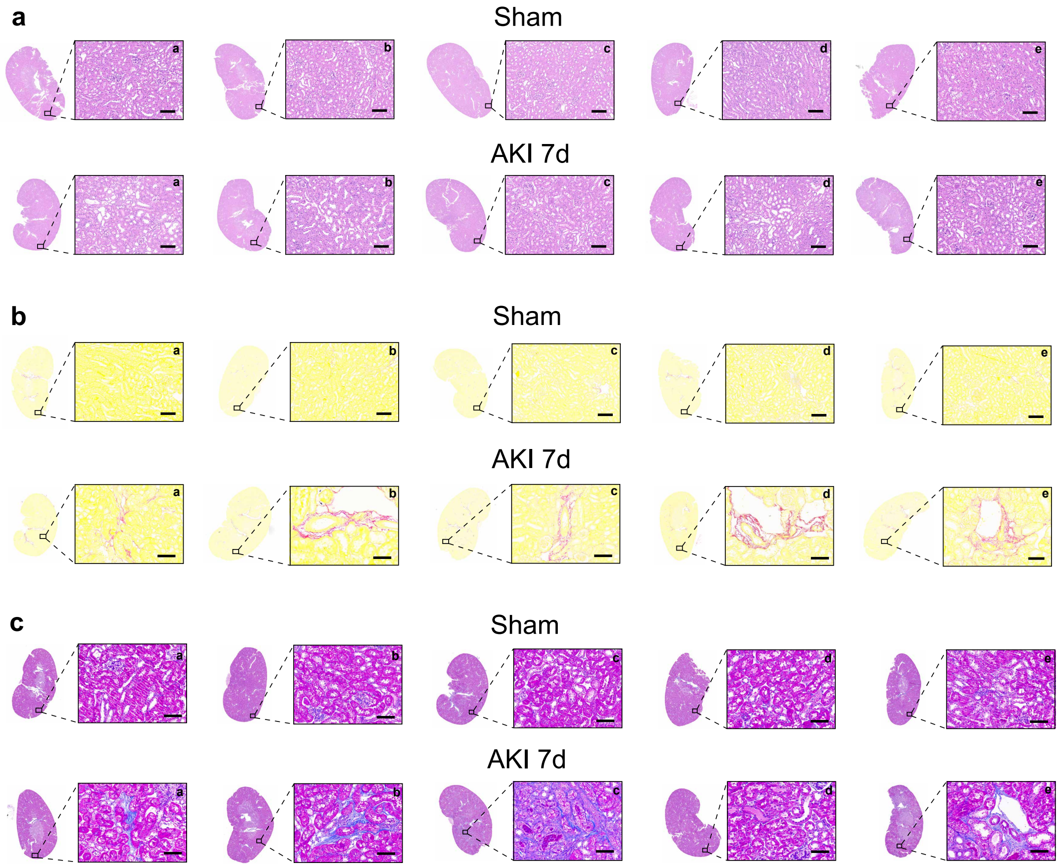

Supplementary Fig. 2. Histological analysis of kidneys from AKI and Sham controls.

(a) H&E staining of kidney sections highlighting ischemic pathological alterations at day 7 post-AKI. **(b)** PSR staining shows fibrotic regions in AKI kidney. **(c)** Masson staining further confirms interstitial fibrosis in AKI kidneys. Scale bar: 50 μm.
