# Supplementary material for: Single-cell transcriptomic analysis reveals the association of Ccl6+Ccr2+Arg1+ macrophages with renal interstitial fibrosis in AKI
Source: PLoS One. 2025 Sep 15;20(9):e0332026. doi: 10.1371/journal.pone.0332026 (PMC12435735; doi:10.1371/journal.pone.0332026)
Supplement: S3 Fig — (PDF) [file pone.0332026.s003.pdf]

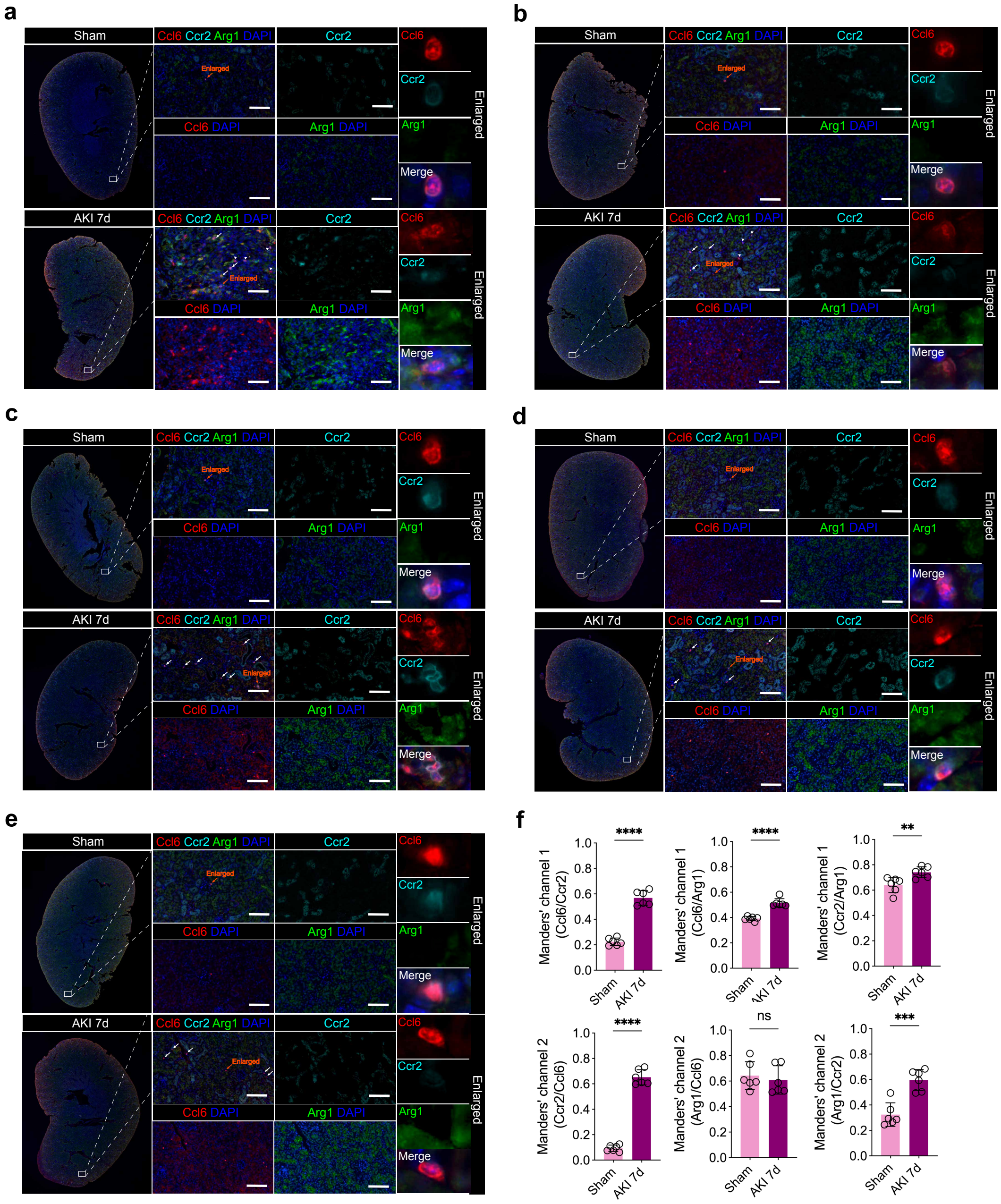


**Supplementary Fig. 3. Multiplex immunofluorescence reveals the co-localization of Ccl6, Ccr2, and Arg1 in macrophages within kidneys 7 days post-AKI.**

**(a, b, c, d, e)** Localization of Ccl6, Ccr2, and Arg1 in kidney sections. Enlarged views highlight co-localization of these markers within macrophages, indicating M2 polarization. Scale bar: 50 µm. **(f)** Immunofluorescence-based quantification showed significantly increased Manders' overlap coefficients among the three markers, indicating enhanced co-expression within the same cells (n = 6).
