# Supplementary material for: Single-cell transcriptomic analysis reveals the association of Ccl6+Ccr2+Arg1+ macrophages with renal interstitial fibrosis in AKI
Source: PLoS One. 2025 Sep 15;20(9):e0332026. doi: 10.1371/journal.pone.0332026 (PMC12435735; doi:10.1371/journal.pone.0332026)
Supplement: S4 Fig — (PDF) [file pone.0332026.s004.pdf]

a

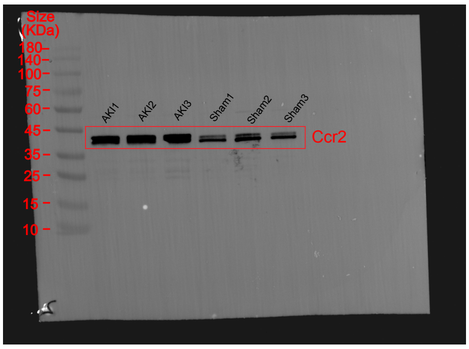

b

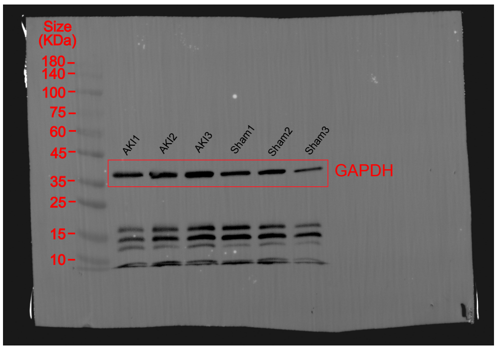

Supplementary Fig. 4. Western blot analysis of Ccr2 expression in AKI kidneys.

(a, b) Representative Western blot images of Ccr2 and GAPDH protein levels.
