# Supplementary material for: Single-cell transcriptomic analysis reveals the association of Ccl6+Ccr2+Arg1+ macrophages with renal interstitial fibrosis in AKI
Source: PLoS One. 2025 Sep 15;20(9):e0332026. doi: 10.1371/journal.pone.0332026 (PMC12435735; doi:10.1371/journal.pone.0332026)
Supplement: S5 Fig — (PDF) [file pone.0332026.s005.pdf]

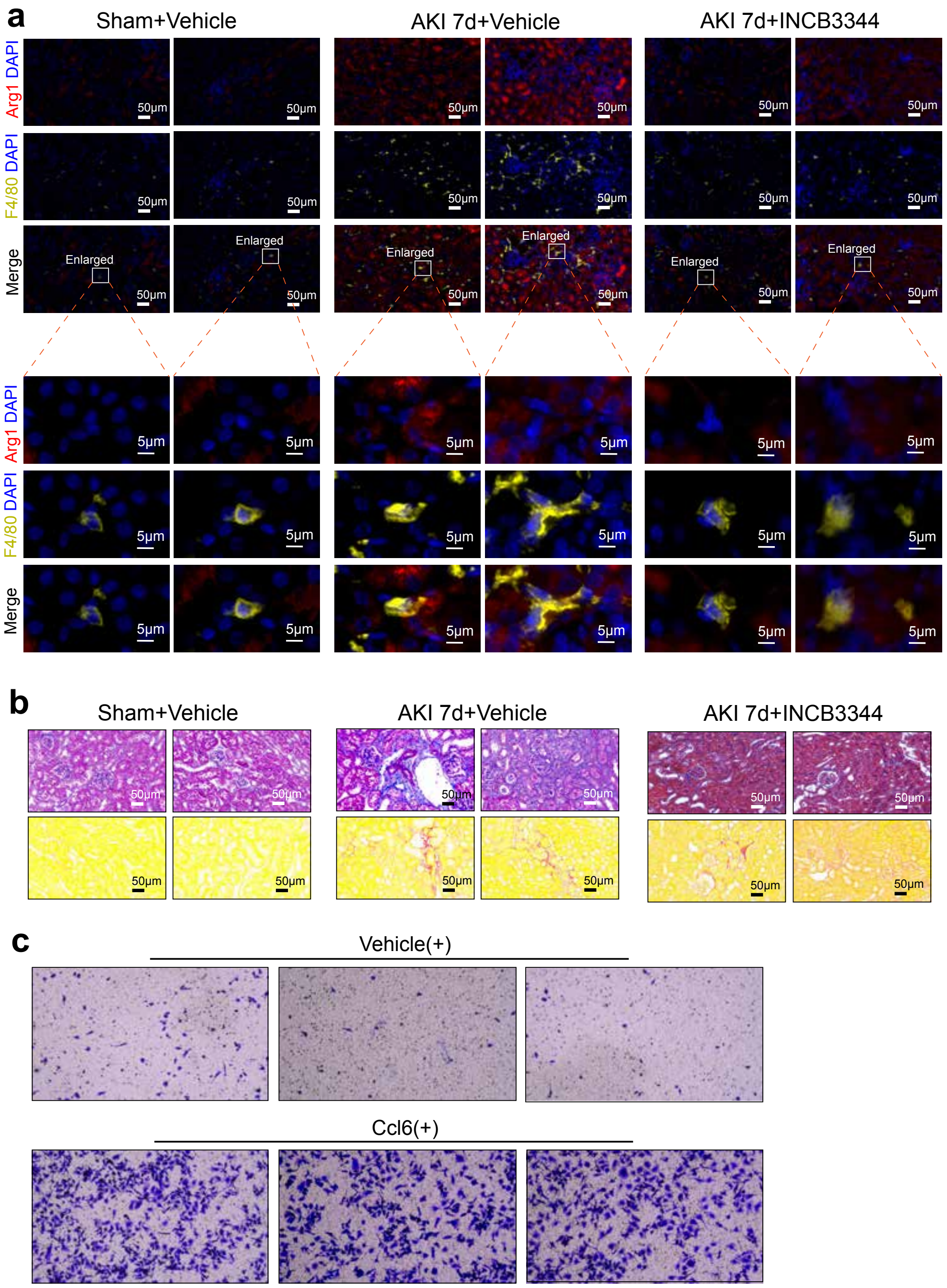

Supplementary Fig. 5. Ccl6/Ccr2 axis mediates macrophage recruitment, M2 polarization, and fibrosis After AKI.

(a) Immunofluorescence results showed that compared with the Sham group, Arg1 and F4/80 expression in AKI kidneys was significantly increased on day 7, but markedly decreased after Ccr2 inhibition. Scale bar: 50 μm. **(b)** Inhibition of Ccr2 significantly reduced collagen deposition and alleviated renal fibrosis compared with untreated AKI controls (n=3). **(c)** Ccl6 stimulation for 24 hours significantly enhanced macrophage migration compared with vehicle-treated controls, indicating its chemotactic effect on BMDMs.
