# Supplementary material for: Single-cell transcriptomic analysis reveals the association of Ccl6+Ccr2+Arg1+ macrophages with renal interstitial fibrosis in AKI
Source: PLoS One. 2025 Sep 15;20(9):e0332026. doi: 10.1371/journal.pone.0332026 (PMC12435735; doi:10.1371/journal.pone.0332026)
Supplement: Attachment — Submitted filename: PONE-D-25-18641_reviewe.docx [file pone.0332026.s007.docx]

This manuscript presents an interesting and timely investigation into the role of a specific macrophage subpopulation in the transition from acute kidney injury (AKI) to fibrosis. The authors combine an integrated analysis of public single-cell RNA sequencing datasets with in vivo validation in a murine unilateral ischemia-reperfusion injury (uIRI) model. The identification of a Ccl6+Ccr2+Arg1+ macrophage population that correlates with the degree of interstitial fibrosis is a novel finding and points to a potentially important cellular player in this pathological process. The manuscript is well-structured and the data are generally presented clearly. However, the study's conclusions are largely based on correlational evidence, and a significant number of points require clarification and further experimental support to substantiate the claims regarding the functional role of the Ccl6/Ccr2 axis in driving fibrosis.

**Major revisions are required:**

- The central claim is that the Ccl6/Ccr2 axis is associated with, and may promote, renal fibrosis. However, the evidence provided is correlational, demonstrating an association between the number of Ccl6+Ccr2+Arg1+ cells and the extent of fibrosis. To move from correlation to causation and truly test the functional significance of this axis, have you considered functional experiments? For instance, in vivo administration of a Ccr2 antagonist or a neutralizing antibody for Ccl6 in the uIRI model would be required to assess whether blocking this interaction attenuates M2 macrophage infiltration and subsequent fibrosis.
- Similarly, can the proposed mechanism be validated in vitro? For example, could bone marrow-derived macrophages (BMDMs) be treated with recombinant Ccl6 protein to assess its effect on macrophage migration, proliferation, or polarization towards an Arg1+ phenotype? This would provide direct evidence for the signaling function of Ccl6 on macrophages.
- The single-cell analysis integrates multiple public datasets. While Harmony was used for batch correction, the robustness of the key findings could be strengthened. Could you please show whether the core discoveries—specifically the upregulation of the CCL signaling pathway and the Ccl6/Ccr2 ligand-receptor pair—are consistently observed across the individual AKI datasets, or if the signal is disproportionately driven by one or two of the datasets?
- The CellChat analysis identifies macrophages as both the primary source of Ccl6 and a key target via Ccr2. The manuscript suggests this drives macrophage migration. Could you clarify whether this interaction is interpreted as being predominantly autocrine (macrophages stimulating their own migration) or if there is also evidence of a paracrine loop where resident macrophages recruit circulating Ccr2+ monocytes? The distinction is mechanistically important.
- The study identifies the Ccl6+Ccr2+Arg1+ population. Using your scRNA-seq data, could you provide a more comprehensive molecular signature for this specific triple-positive subset? What other pro-fibrotic genes (e.g., Spp1, Lgals3, Tgfb1) or matrix-remodeling enzymes are co-expressed in these cells compared to Arg1+ cells that are negative for Ccl6/Ccr2? This would offer a more granular view of their potential function.
- The references cited in this article are not sufficient, and there is a lack of in-depth comparative discussion. Background and methodology also require further literature support. Some related research should be cited:

10.15212/bioi-2024-0052

10.15212/bioi-2024-0037

10.15212/bioi-2024-0062

10.1093/burnst/tkac059

10.1093/burnst/tkac052

10.15212/CVIA.2024.0051

10.34133/research.0063

10.5847/wjem.j.1920-8642.2023.057

- In the multiplex immunofluorescence analysis (Figure 5d), the claim of identifying triple-positive cells relies on visual assessment of merged images. To make this conclusion more robust, please provide higher magnification images and, critically, quantitative colocalization analysis (e.g., using Pearson's correlation coefficient or Manders' overlap coefficient) to statistically validate the co-expression of all three markers within single cells.
- The manuscript structure is logical, but the Discussion section could be strengthened. While the limitations are appropriately acknowledged, the discussion would benefit from a deeper exploration of the potential downstream pathways activated by Ccl6/Ccr2 signaling in macrophages that could lead to a pro-fibrotic phenotype.
- Please review the manuscript for professional language and consistency. For example, the heading "Ccl6 and Ccr2 interaction induced macrophage autologous migration" uses the term "autologous migration," which is non-standard. "Autocrine-mediated migration" or rephrasing to "The Ccl6-Ccr2 interaction promotes macrophage migration" would be more appropriate. Please ensure consistent use of terminology throughout.
- The conclusions drawn in the Abstract and Conclusion section should be carefully worded to accurately reflect the data. As the study is currently correlational, phrases like "Targeting the Ccl6/Ccr2 axis may attenuate fibrotic progression", while plausible, should be presented as a hypothesis to be tested in future functional studies, rather than a direct conclusion from the present work.
